# Learning pair-wise gene functional similarity by multiplex gene expression maps

**DOI:** 10.1186/1471-2105-13-S3-S1

**Published:** 2012-03-21

**Authors:** Li An, Haibin Ling, Zoran Obradovic, Desmond J Smith, Vasileios Megalooikonomou

**Affiliations:** 1 Data Engineering Laboratory, Department of Computer and Information Sciences, Temple University, PA, USA; 2 Center for Data Analytics and Biomedical Informatics, Temple University, PA, USA; 3 Department of Molecular and Medical Pharmacology, David Geffen School of Medicine, UCLA, CA, USA

## Abstract

**Background:**

The relationships between the gene functional similarity and gene expression profile, and between gene function annotation and gene sequence have been studied extensively. However, not much work has considered the connection between gene functions and location of a gene's expression in the mammalian tissues. On the other hand, although unsupervised learning methods have been commonly used in functional genomics, supervised learning cannot be directly applied to a set of normal genes without having a target (class) attribute.

**Results:**

Here, we propose a supervised learning methodology to predict pair-wise gene functional similarity from multiplex gene expression maps that provide information about the location of gene expression. The features are extracted from expression maps and the labels denote the functional similarities of pairs of genes. We make use of wavelet features, original expression values, difference and average values of neighboring voxels and other features to perform boosting analysis. The experimental results show that with increasing similarities of gene expression maps, the functional similarities are increased too. The model predicts the functional similarities between genes to a certain degree. The weights of the features in the model indicate the features that are more significant for this prediction.

**Conclusions:**

By considering pairs of genes, we propose a supervised learning methodology to predict pair-wise gene functional similarity from multiplex gene expression maps. We also explore the relationship between similarities of gene maps and gene functions. By using AdaBoost coupled with our proposed weak classifier we analyze a large-scale gene expression dataset and predict gene functional similarities. We also detect the most significant single voxels and pairs of neighboring voxels and visualize them in the expression map image of a mouse brain. This work is very important for predicting functions of unknown genes. It also has broader applicability since the methodology can be applied to analyze any large-scale dataset without a target attribute and is not restricted to gene expressions.

## Background

Functional genomics studies gene functions on a large scale by conducting parallel analysis of gene expression for a large number of genes [1]. The Gene Ontology (GO) represents an important knowledge resource for describing the function of genes [2] and has been widely used for identifying functional similarities [3,4]. A lot of research has been done to explore the relationship between the GO-based similarity and gene expression profiles [5-8], and also the relationship between gene function annotation and gene sequence [9]. All these studies are based on the assumption that genes with similar functions have similar expression profiles in cells [10] or have similar gene sequences. However, little research considers the relationship between the gene functions and the location of a gene's expressions in the mammalian tissues. Voxelation is a new approach that involves dicing the brain into spatially registered voxels (cubes) and performing microarray experiments in each voxel to detect expression values of a large number of genes. Combining voxelation with microarray analysis produces multiple volumetric maps of gene expression values of the mice brain. The expression maps are analogous to the images reconstructed in biomedical imaging systems [11-13] and show good agreement with known expression patterns [14].

Different machine learning methods have been proposed to analyze gene expression data and predict gene functions. The most popular methodologies in functional genomics are unsupervised learning methods such as clustering algorithms, which use a similarity measure to cluster genes with similar expression profiles [15]. Previously we identified the similarity of gene expression maps using the wavelet transform and the similarity between gene functions based on the GO structure and appropriate distance measures [14]. We also performed unsupervised learning methods, like clustering analysis, on the voxelation dataset to detect gene clusters that have both similar gene expression maps and similar gene functions [14]. However, by only having the gene expression patterns of normal genes, it is hard to directly use supervised learning methods for mining biological rules from gene expression maps. In this study, we build a new dataset of pair-wise genes, so that the supervised learning methods, like classification and regression, can be applied. We extract more features besides the wavelet features, and further study the relationship between the gene maps and functions based on the new dataset. Although there are similar studies which use pairs of genes to predict gene functions [16,17], these studies are based on a small number of genes, while our methods are good at analyzing a huge number of genes and identifying the significant voxels in the mice brain.

In this paper we introduce an approach to identify pair-wise gene functional similarities from gene expression maps employing supervised learning techniques. A new dataset is formed by considering pairs of genes from the voxelation dataset as samples. For each sample gene pair, the similarities or distances between the corresponding gene expression maps are used as features to describe it. The labels for gene pairs are their functional similarities. Consequently, we formulate the problem of identifying the functional similarity between genes as a supervised learning problem. We use AdaBoost as the basic framework for our learning and prediction task. In order to fit the dataset which has huge number of samples and limited number of features, we propose a novel weak classifier that efficiently captures the distribution of individual features. We further restrict the dataset to the genes which are associated with previously detected functional expression profiles to strengthen the relationship between gene functions and gene maps. The experimental results show that the pair-wise gene functional similarities are increased with increasing similarities of gene expression maps. In addition, the boosting analysis classifies, with a high accuracy, the gene pair samples into two classes: pairs of genes with similar functions and those without. The analysis of feature selection in the learning process indicates which features are significant for identifying the functional similarity from gene expression maps. Those features can be located and visualized in the expression map image of a mouse brain. These findings can be potentially used for predicting gene functions and providing helpful clues to biologists. This manuscript significantly adds to the preliminary results reported in [18] by investigating new image features, performing new experiments to select the most significant of the 455 features, analyzing more gene ontologies, and performing regression in addition to classification used earlier [18]. The most significant features detected are different and more effective from those obtained in [18] resulting in increased prediction accuracy.

The rest of this paper is organized as follows. In the methods section, after describing the pair-wise samples of multiple gene expression maps and briefly discussing how to extract features from the original gene expression maps and identify gene function distance, we present approaches for identifying functional similarities of pair-wise samples by Boosting and our proposed weak classifier. In the results section, we present the experimental results of identifying the relationship between similarity of gene expression maps and their functions, as well as the results from boosting analysis. The discussion section provides an analysis of the obtained results and ideas for future applications of this methodology.

## Methods

### Gene expression maps

**Figure 1:**
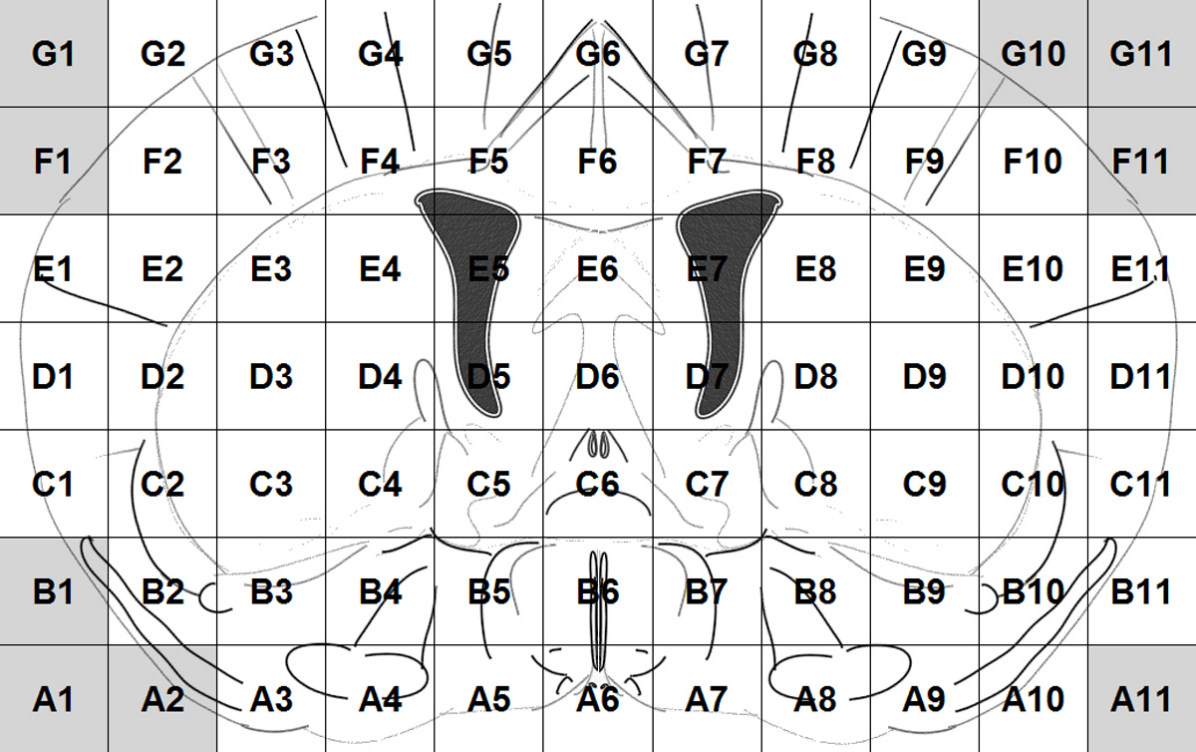
Voxels of the coronal slice.

**Figure 2:**
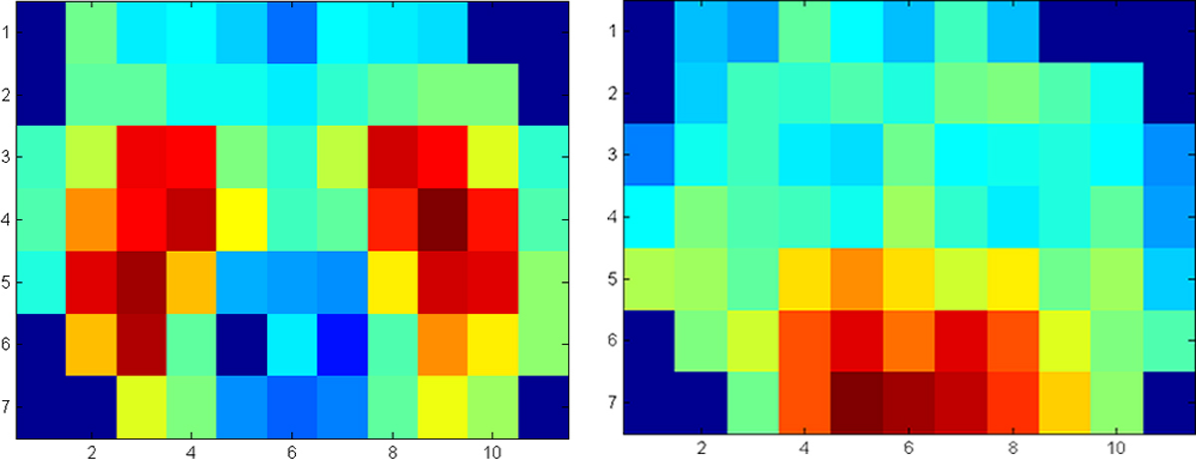
Visualized gene expression maps.

Voxelation has been used in combination with microarrays for acquisition of genome-wide atlases of expression patterns in the mouse brain [13]. For this study multiplex gene expression maps for 20,847 genes have been acquired using the procedure below. After obtaining a 1 mm coronal slice of the brain at the level of striatum the slice is cut by a matrix of blades resulting in cubes (voxels) that are 1 mm3. The locations of these voxels on the slice are recorded as Figure 1 shows. Voxels A1, A2, B1... are in gray because they correspond to empty cubes that are assigned to maintain a rectangular. So, each gene is represented by the gene expression values of 68 voxels that compose a gene expression map of a mouse brain. By using different colors to show different values of gene expression, the expression map of the genes can be visualized as in Figure 2.

The dataset we consider in this study forms a 20,847 by 68 matrix, where each row represents the log2 ratio 68 expression values of a particular gene, and each column represents the expression values for all the probes (genes) at a given voxel. To reduce the effects of noise in the original dataset, we discard genes whose gene expression values fall in the range [-1,1]. The remaining 13,576 genes' IDs are imported into the SOURCE [19] database [20] to retrieve their Gene Ontology (GO) annotation information. Out of the 13,576 genes, 7,883 genes are known genes and are annotated with at least one GO term. Our analysis is based on these genes. We denote the set of genes as G={g_1_, g_2_,..., g_N_}, where N = 7,883.

### Pair-wise samples

Gene expression maps can be viewed as samples that can be analyzed using data mining techniques. However, the targets or labels associated with each sample are not always available, as is the case in our study. Therefore, we form a new dataset by considering each pair of gene expression maps as a sample, and calculating the functional similarity of the gene pair i.e., the distance between the functions of its two genes, which becomes the label for that sample. As a result, the problem of identifying the relationship between gene expression maps and gene functions is formulated as a regression problem. That is, a sample is defined as

$${\text{(g}}_{\text{1}},{\text{ g}}_{\text{2}})\text{ with a }“\text{label}”{\text{ d}}_{\text{F}}({\text{g}}_{\text{1}},{\text{g}}_{\text{2}}),$$

where (g_1_,g_2_) is a pair of genes, and d_F_(g_1_,g_2_) is the desired function distance for this pair of genes which we intend to approximate.

Suppose g_1_, g_2_, g_3_,... are gene maps and d_F_(g_1_, g_2_), d_F_(g_2_, g_3_), d_F_(g_1_, g_3_) are gene function distances between the pairs (g_1_, g_2_), (g_2_, g_3_), (g_1_, g_3_) respectively, we have samples for all the gene pairs:

$${\text{g}}_{1},\;{\text{g}}_{2}\;{\text{d}}_{\text{F}}\left({\text{g}}_{\text{1}},\;{\text{g}}_{\text{2}}\right)$$

$${\text{g}}_{1},\;{\text{g}}_{3}\;{\text{d}}_{\text{F}}\left({\text{g}}_{\text{1}},\;{\text{g}}_{\text{3}}\right)$$

$${\text{g}}_{2},\;{\text{g}}_{3}\;{\text{d}}_{\text{F}}\left({\text{g}}_{\text{2}},\;{\text{g}}_{\text{3}}\right)$$

Given this dataset, the problem boils down to finding the relation between the pair-wise gene expression map similarity and the pair-wise gene functional similarity. In our previous analysis of gene expression maps [14], we have defined the similarity between two gene expression maps as the Euclidean distance between their wavelet representations, and calculated the similarity (distance) between two gene functions based on gene ontology structures using Lin's method [21]. We have shown that the similarity between gene expression maps is positively correlated to the similarity between gene functions, which encourages the study of the relationship between pairs of gene maps and their functional similarity.

### Functional similarity of pairs of genes

We perform the analysis with respect to each one of the three gene ontologies, i.e., cellular component, molecular function and biological process. For example, in the category of biological process, if gene g_1 _has functions F(g_1_) ={f_11_, f_12,...,, _f_1n_} and gene g_2 _has functions F(g_2_) = {f_21_, f_22,..., _f_2 m_}, we define the function similarity (or distance) value between these two genes as the averaged functional distance of pairs of functions between the two genes. This is calculated using the following formula:

$$d_{F}\left(g_{1},g_{2}\right)={\left\{\begin{array}{cc} \frac{1}{\Gamma }\underset{f_{1}\in F\left(g_{1}\right)}{\sum }\underset{f_{2}\in F\left(g_{2}\right)}{\sum }d_{func}\left(f_{1},f_{2}\right) & \;\;if\;\Gamma >0\\ \begin{array}{c} 0 \end{array} & \begin{array}{c} \;\;\\ \;\;if\;\Gamma =0 \end{array} \end{array}\right)}_{},$$

where

$$\Gamma =\#\left\{d_{func}\left(f_{1},f_{2}\right)>0,\forall f_{1}\in F\left(g_{1}\right),f_{2}\in F\left(g_{2}\right)\right\}$$

provides a count of the number of function pairs with non-zero distances and *d_func_*(.,.) is the gene function distance.

### Extracting features from the expression maps and forming the feature vector

**Figure 3:**
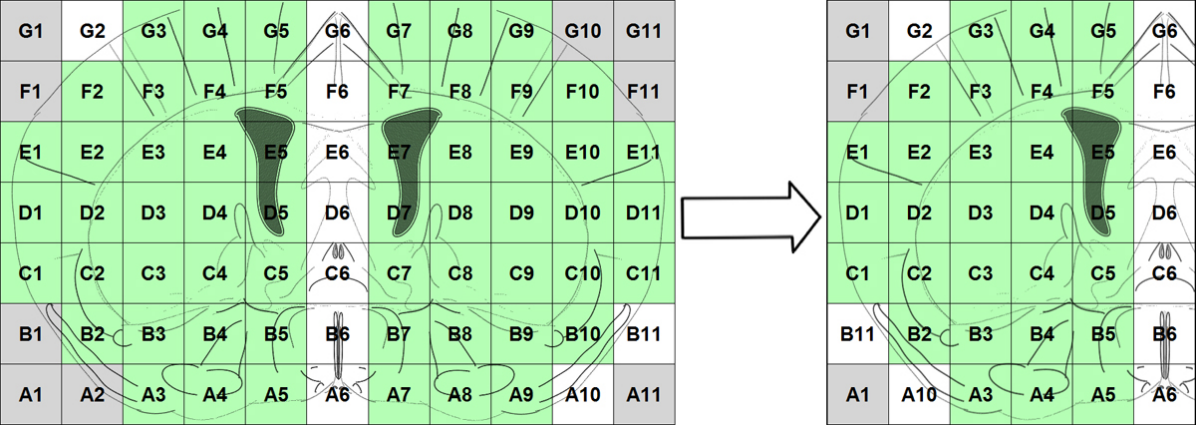
Averaging left and right hemispheres of the mouse brain.

First, in order to reduce the noise in microarray experiments and improve the signal, we average the left and right hemispheres by taking advantage of the inherent bilateral symmetry of the mice brain. For each row of the map, we average the green cells, as shown in Figure 3, replace B1 with B11, A2 with A10, and the averaged gene expression map is obtained. In order to take into account the spatial location of voxels in the brain map, we use the wavelet transform to extract features from the averaged gene expression map (right part of Figure 3). By employing multilevel 2-D wavelet decomposition at level 3, we obtain 42 coefficients, i.e. wavelet features. More detailed information on extracting the wavelet features is given in [14].

In addition to the 42 wavelet features and the 68 original expression values, we introduce three new features: the correlation coefficient, the p-value of the correlation coefficients, and the Euclidean distance between pair-wise gene maps for each sample. Each p-value is the probability of getting by chance a correlation as large as the observed value, when the true correlation is zero. If P(i,j) is small, e.g., less than 0.05, then we consider the correlation R(i,j) to be significant.

**Figure 4:**
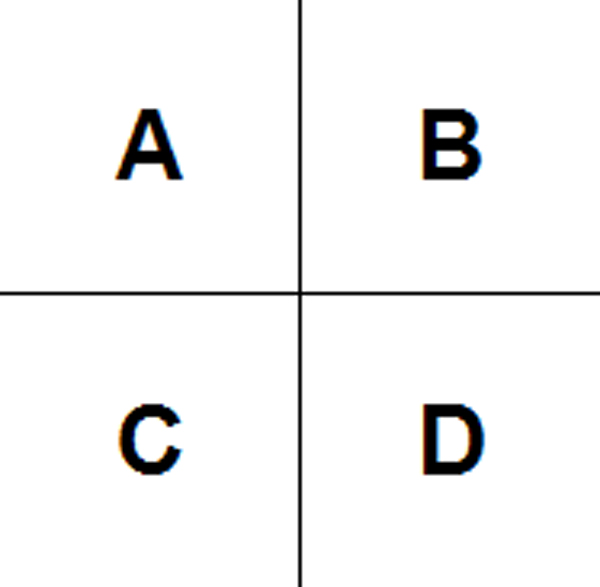
Examples of pairs of neighbouring voxels.

Moreover, we extract features from pairs of neighboring voxels in the gene expression maps. The neighboring voxels, for example, in Figure 4, include the horizontal pairs of cells (A, B) and (C, D), the vertical pairs of cells (A, C) and (B, D), and the diagonal pairs of cells (A, D) and (C, B). Among the 68 cells in Figure 1, there are 61 pairs of horizontal neighboring cells, 57 pairs of vertical neighboring cells, and 53 pairs of diagonal neighboring cells. So, we have a total of 171 (= 61 + 57 + 53) pairs of neighboring voxels. For each pair, we average the gene expression values and calculate the absolute value of the difference of the two cells. For example, for the pair of voxels (A, B), the average value is (A+B)/2 and the absolute value of difference is |A-B|. Thus, we totally extract 342 (= 171*2) features from the pairs of neighboring voxels.

Therefore, for each gene map, we concatenate its 42 wavelet coefficients, the 68 gene expression values, the three new features, and the 342 features of the neighbouring voxels, resulting in a 455 dimensional descriptor. Given two genes g_1 _and g_2_, let *W*_1 _and *W*_2 _be their feature vectors respectively. We derive the feature vector *V *of the gene pair (*g*_1_, *g*_2_) such that

$$V\left(\text{i}\right)=|W_{\text{1}}\left(i\right)-W_{\text{2}}\left(i\right)|$$

Therefore, a gene pair sample can be represented as:

$$\left(V,\;d_{F}\left(g_{\text{1}},g_{\text{2}}\right)\right).$$

### Identifying functional similarities of pair-wise samples by boosting

Having the features and the samples ready, we need to choose a learning technique for our task. Here we face the challenge of dealing with a huge sample dataset. There are in total 31,066,903(= 7883 × 7882/2) samples of gene pairs. Each sample has 110 features. The dataset is too large to be handled by many popular machine learning methods, such as the Support Vector Machine. Boosting [22], however, solves this problem by loading and computing samples and features (weak learners) sequentially. Another advantage of using boosting is that it provides a way to investigate the roles of features in the learned classifier/regressor. In our particular task, this helps understanding the importance of each individual feature in predicting the gene similarities.

Since boosting is usually used to solve classification problems, we need to transform the regression problem to a classification problem by setting a threshold. The threshold is used to classify the continuous values of function distances into two classes: one that includes the samples (pairs of genes) with similar functions, and another that includes the samples with non-similar functions. So the classification problem with the continuous output in the range [-1,1] is transformed to a problem with two classes {-1, 1}, through a predefined threshold.

There are several variants of boosting algorithms that are widely used in the fields of data mining and pattern recognition. We choose AdaBoost [23] due to its excellent performance observed in many applications and its flexibility in weak classifier design. Intuitively, AdaBoost uses a weighted additive model to fit the training data. The model, which is named a *strong *classifier, is a weighted summation of a set of *weak *classifiers. The weight and weak classifiers are iteratively estimated or selected until convergence.

In our task, for an input feature vector V, a strong classifier denoted as H(V) is formulated as a combination of weak classifiers h_1_(V), h_2_(V),..., h_K_(V):

$$H\left(V\right)=\overset{K}{\underset{k=1}{\sum }}c_{k}h_{k}\left(V\right)\;,$$

where c_k _is the weight for the k-th weak classifier h_k_. The task of the learning process is, in the k-th iteration where k = 1,...K, to either fit h_k _or to pick h_k _from a candidate set of weak classifiers. The fitting or selection is based on the classification performance achieved on the training samples weighted by the current weights.

### Designing the weak classifiers

One popular way of designing weak classifiers is to associate with each weak classifier a threshold in order to create a binary classifier, i.e., a stump function. In particular, for the i-th feature V(i) in our feature vector V and a threshold τ, a weak classifier has the form

$$h^{i,\tau }\left(V\right)=\left\{\begin{array}{cc} 1 & \;\;if\;V\left(i\right)\leq \tau \\ -1 & \;\;if\;V\left(i\right)<\tau \end{array}\right).$$

The learning process is to find i, τ, and c_k _for each one of the weak classifiers. In this case, a weak classifier is associated with only one feature. As a result, the weight c_k _can be used to evaluate the importance of the feature in the strong classifier, i.e., the ultimate model used for prediction.

The binary classifier is very simple and easy to implement. However, for a complex learning task such as the one we are dealing with, having weak classifiers that are more effective often helps improving the learning and predicting efficiency while reducing the number of weak classifiers needed. In addition, in our study we have a huge set of training samples, which enables us to use better but more complex weak classifiers. Motivated by this observation, we extend the simple stump classifier by modeling the weak classifier with uniformly spaced bins. Specifically, our weak classifier for the i-th feature contains an *indicating *vector L∈{-1,1}^M^, where M is a predefined number of bins. A classifier has the following form

$$h^{i,L}(V)=L(index(V(i))\;,$$

where index(V(i)) is the index of the bin V(i) falls into. In the learning stage, the task at each iteration is to select the feature i that best estimates the indicating vector L. This is done by building a cumulated weight followed by a voting. The stump weak classifier can be viewed as a simplified case where M = 2.

**Figure 5:**
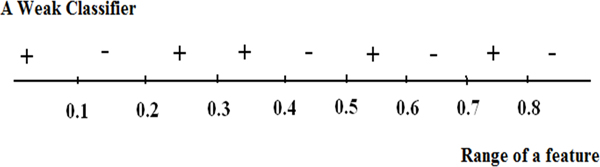
An example of a proposed weak classifier.

Figure 5 shows an example of a weak classifier learned from one of the features of the training data. It shows that the range of features is divided into small regions. The intervals of weak classifiers depend on the range of each feature (i.e. the max and min values of the feature). We divide the range uniformly with fixed sizes. The label for each region is the sum of weighted labels of samples within the region. When the weak classifier is used in prediction, the sample is assigned the label of the region in which this specific feature falls.

### Applying the learning method to the MFEP specific subset

The relationship between the gene functions and gene expression maps does not hold for all genes but only for a certain set of genes. For this reason, we take advantage of previous results obtained [24] using *multiple functional expression profiles *(MFEPs) to perform the boosting analysis. For a given gene function or a set of gene functions, there might be a specific gene expression map (profile) associated with it. Genes that have similar gene expression maps to a specific profile may hold similar gene functions. We call this specific gene expression profile for a set of functions, Multiple Functional Expression Profile (MFEP). Genes associated to an MFEP have the same set of gene functions and also have very similar gene expression maps. The detected MFEPs can be used to predict gene functions with high accuracy [24]. In order to explore the strong relationship between gene functions and expression maps, we use a subset of genes instead of the whole dataset. This subset is created by calculating the expression features and labels of pairs of genes that are associated with the detected MFEPs, so we call it *MFEP specific subset*. Using this subset, we aim to discover a regression relationship between the gene expression similarity (expression features) and gene function similarity (labels).

### Performing regression using boosting on the MFEP specific subset

For the regression, the label of each sample is taken as the functional similarity between a pair of genes which is a continuous number in the range of 0 to 1. The regression is performed by the AdaBoost algorithm. To create the weak classifier, a threshold is again used to divide the dataset into two classes, with each sample having a label of -1 or 1. As the weighted summation of weak classifiers, the outputs of the strong classifier are fed into logistic transformation to produce regression results in the range of 0[1]. The modified AdaBoost algorithm to do the regression is given below [23]:

Given: (*x*_1_,*y*_1_),...,(*x_m_,y_m_*), where *x*_i_∈*X y*_i_∈*Y *= {-1, +1}

Initialize $D_{1}\left(i\right)=\frac{1}{m},\;i=1,...,m$

For *t *= 1,...,T:

Find the classifier *h_t_: X*→{-1,+1} that minimizes the error with respect to the distribution *D_t_*:

$h_{t}=\text{arg}\underset{h_{j}\in H}{\text{min}}\varepsilon_{j}$, where $\varepsilon_{j}=\overset{m}{\underset{i=1}{\sum }}D_{t}\left(i\right)\left[y_{i}\neq h_{j}\left(x_{i}\right)\right]$

if *ε_t _*> = 0.5 then stop.

Choose *α_t_*∈*R*, typically $\alpha_{t}=\frac{1}{2}\text{ln}\frac{1-\varepsilon_{t}}{\varepsilon_{t}}$, where *ε_t _*is the weighted error rate of classifier *h_t_*.

Update:

$$D_{t+1}\left(i\right)=\frac{D_{t}\left(i\right)\text{exp}\left(-\alpha_{t}\cdot y_{i}\cdot h_{t}\left(x_{i}\right)\right)}{Z_{t}}$$

where *Z_t _*is a normalization factor (chosen so that *D*_*t *+ 1 _becomes a probability distribution, i.e. sum one over all elements).

Output the final classifier:

$$\underset{f}{\text{Pr}}\left[y=+1|x\right]=\frac{e^{f\left(x\right)}}{e^{f\left(x\right)}+e^{-f\left(x\right)}}$$

Where *f*(*x*) is the weighted average of base classifiers produced by AdaBoost

$$f\left(x\right)=\sum_{t}\alpha_{t}h_{t}\left(x\right)$$

## Results

Based on the proposed methodologies, we study the relationship between similarities of gene expression maps and their functions. We also perform boosting analysis on the MFEP specific and restricted subsets and built models. Using the boosting models, we predict pair-wise functional similarities and calculate the prediction accuracies. At last, we perform regression analysis using the modified Adaboost on the MFEP specific and restricted subsets. The detailed experimental results are presented and analyzed in the following sections.

### Identifying the relationship between similarity of gene expression maps and their functions

First, we analyze the MFEP specific subset which consists of genes associated with the MFEPs. We use the correlation coefficients *R *and associated p-values *P *among the 42 wavelet features to identify the similarity between gene expression maps. Specifically, *R *contains the correlation coefficients between genes and *P *contains the p-values for the hypothesis of no correlation. *R *is taken as the similarity between gene maps to analyze the subset of genes within MFEPs. Given an interval of *R*, for example [0.1, 0.2], we select the set of samples falling within this interval and average their functional similarities.

**Figure 6:**
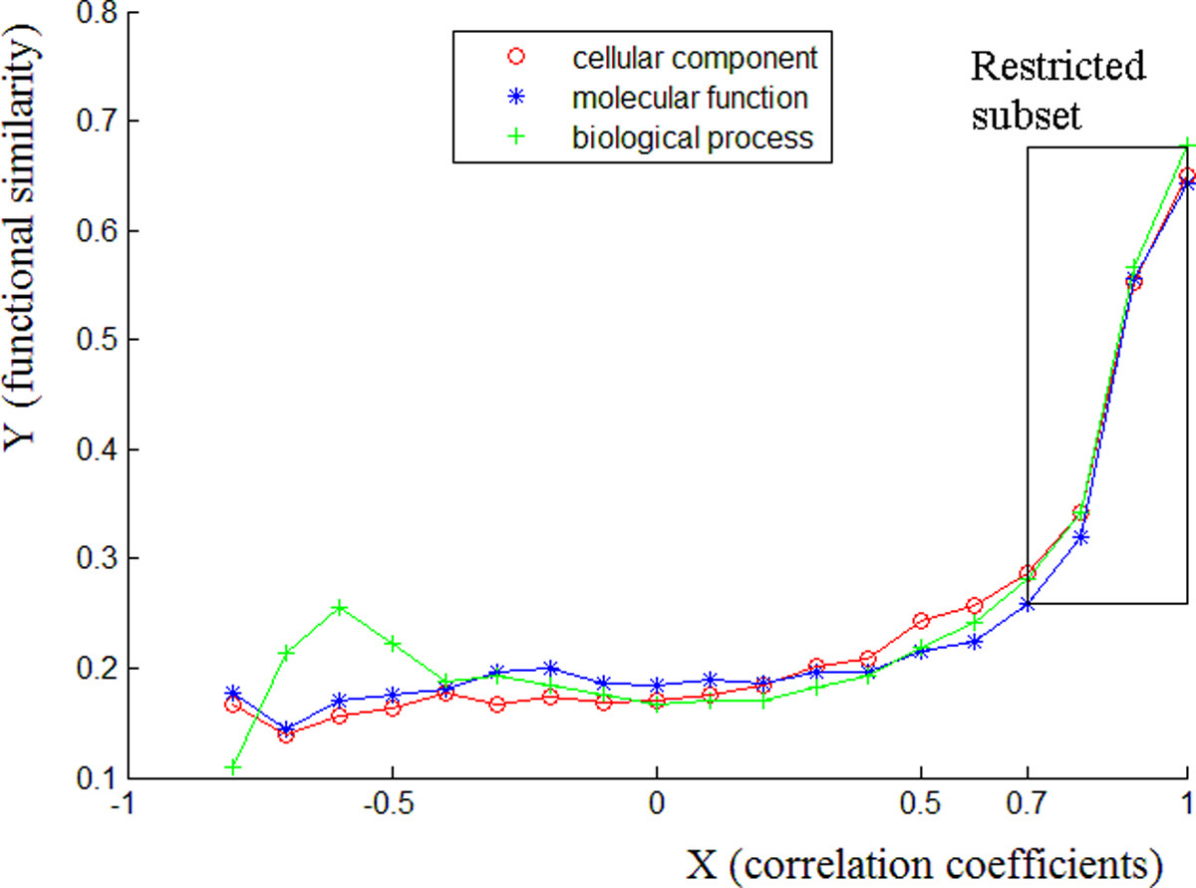
**The distribution of correlation coefficients of gene maps similarity to functional similarity.** X (-1:0.1:1) are the correlation coefficients for pairs of gene expression maps. Y is the averaged functional similarities of the samples whose correlation coefficients (X) are within a certain interval.

All 345 genes associated to the MFEPs are used in the experiment, resulting in the number of combinations of 2 elements from a total of 345 elements, i.e., C(345, 2) (= 59,340) samples. The distribution of the correlation coefficients and the corresponding averaged functional similarities of samples are shown in Figure 6. The figure shows that when the similarities of gene maps are increasing, the function similarities are also increasing. The trend is very obvious for the samples with high correlation coefficients (larger than 0.6).

### Boosting analysis on the MFEP specific subset

We conducted the boosting analysis on the MFEP specific subset of the 59,340 samples using our proposed weak classifiers and AdaBoost. The dataset was randomly split into two disjoint dataset: a training set (29,670 samples) and a test set (29,670 samples). The functional similarities between two genes are continuous values in the range 0[1], where "0" indicates no functional similarity and "1" indicates that the two genes have exactly the same functions. In the experiment, we set the threshold 0.3 as a cut-off value for the similarity. If the value was larger than 0.3, we set the label to 1, otherwise we set the label to -1. With this threshold, there were 33.5% training samples that were assigned label 1, and 33.3% control (test) samples that were assigned label 1. The model was learned based on the training set, and was then used to predict the labels of samples in the test set.

For the weak classifier, we chose the value 20 for the number of bins. For the AdaBoost algorithm, the boosting was repeated for 6000 iterations to reach a stable performance on the prediction. Using these settings, we calculated the prediction error on the training and control samples.

With respect to cellular component, the minimum error on training data was 25.05%, and the minimum error on control data was 29.79%. For the number of iterations performed, the error converged to a certain value. By changing the number of regions and the number of iterations, the error rate varied. The prediction errors with respect to other gene ontologies are shown in Table 1.

**Table 1:** Minimum prediction errors on the MFEP specific and restricted subset with respect to gene ontologies

Ontology	Minimum error on specific subset		Minimum error on restricted subset	
	
	Training data	Control data	Training data	Control data
Cellular Component	25.05%	29.79%	0	16.9%
Molecular Function	32.09%	37.36%	0	25.14%
Biological Process	27.72%	28.84%	0	20.47%

**Figure 7:**
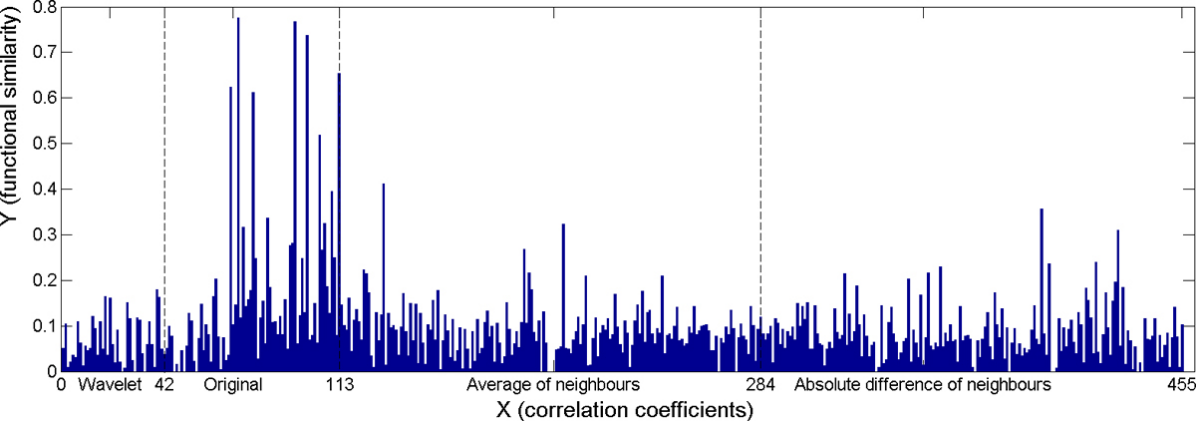
**Cumulated weight of selected features on MFEP specific subset with respect to cellular component.** 1 - 42: wavelet features; 43 - 110: original voxels; 111: the correlation coefficient; 112: the p-value of the correlation coefficients; 113: the Euclidean distance between pair-wise gene maps; 114 - 284: average of neighbouring voxels; 285 - 455: absolute value of difference between neighbouring voxels.

Boosting selects the best feature (weak classifier) at each iteration and gives a weight to the feature. Figure 7 shows the cumulated weight of the 455 features over the 6000 iterations with respect to cellular component. The histogram bar corresponding to a certain feature is the sum of the weights of the feature which are selected during the 4000 iterations. For example, if a feature is selected *m *times with weights w_1_, w_2_,..., w_m_, the sum of weights of the feature is ∑_i = 1_^m ^w_i_. Among the 455 features, the top 10 selected features were the 72^nd^, 95^th^, 100^th^, 113^rd^, 69^th^, 78^th^, 105^th^, 131^st^, 110^th^, and 398^th ^features. Observe that the most selected features were the original expression values (72^nd^, 95^th^, 100^th^, 69^th^, 78^th^, and 105^th^), the average of neighbour cells (131^st^), the absolute value of difference between neighbour cells (398^th^), and the Euclidean distance between pair-wise gene maps. Since the Euclidean distance directly reflects the appearance similarity of two gene maps, this observation strongly supports our conjecture that gene map similarities correlate closely with the gene functional similarities. The wavelet features were not among the top 20 features selected.

**Figure 8:**
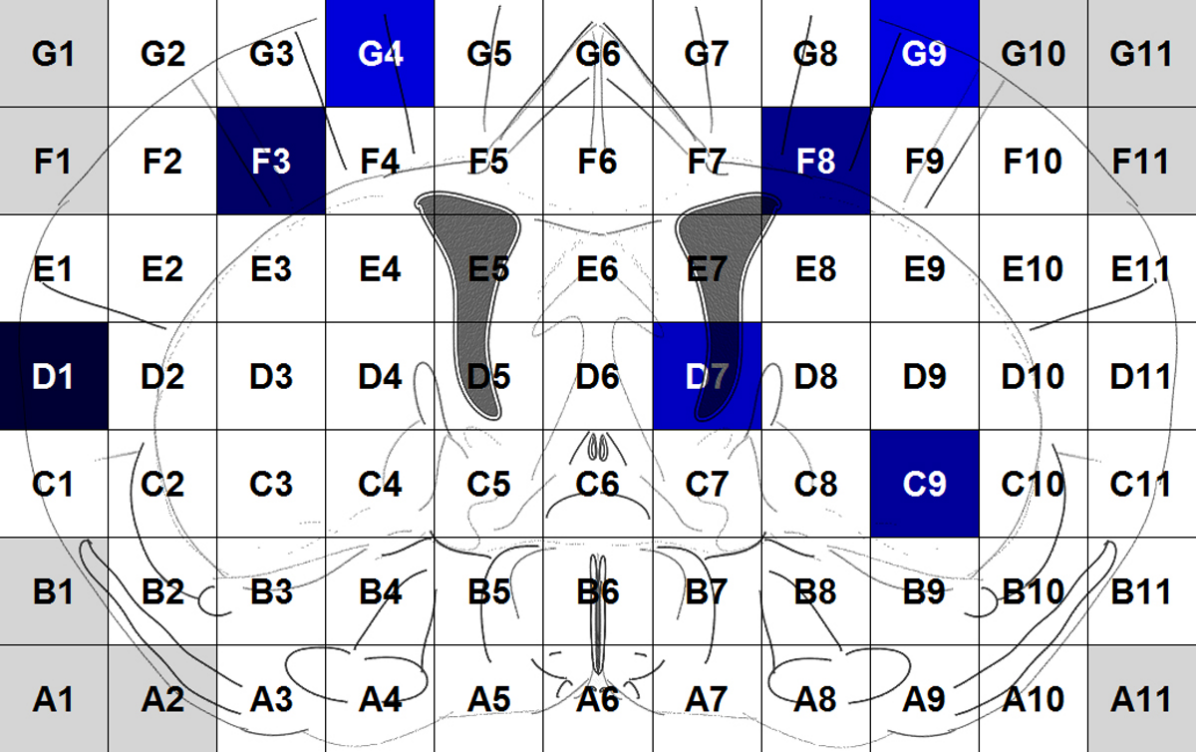
**The most selected original voxels on MFEP specific subset with respect to cellular component (best viewed in color).** The darker mark means that the voxel is more significant.

**Figure 9:**
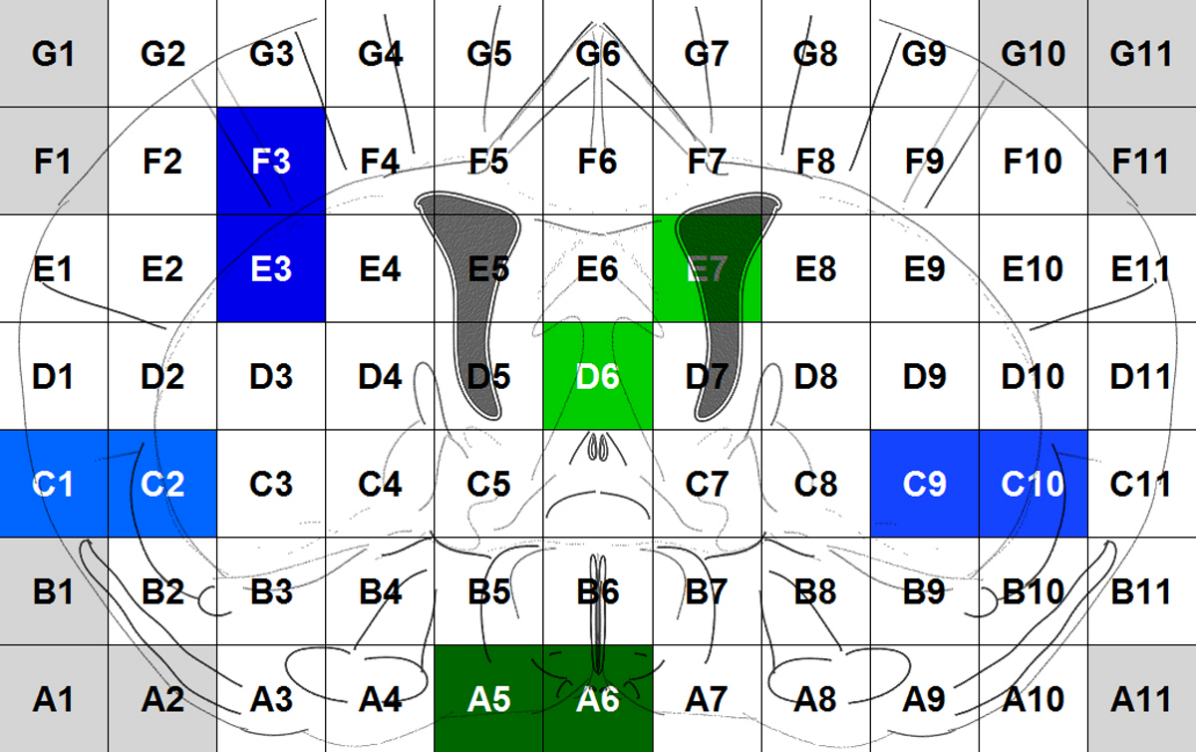
**The most selected wavelet features on MFEP specific subset with respect to cellular component (best viewed in color).** Blue indicates the average expression values of neighbouring voxels; Green indicates the absolute value of difference between neighbouring voxels. The darker mark means that the pairs are more significant.

Because the 68 original features are gene expression values in the 68 voxels (Figure 1), we can visualize and locate these features in the mouse brain. For example, the most selected original voxels with respect to cellular component are shown in Figure 8 as D1, F3, F8, C9, D7, G4, and G9. In the figure, the darker mark indicates that the voxel is selected more frequently (in terms of sum of weights) and that is more significant in predicting the functional similarity of genes from the gene expression maps. The boosting experiment also selected features extracted from pairs of neighbour cells. The top selected such features were the average expression values of pairs of voxels: (F3, E3), (C9, C10), (C1, C2), and the absolute value of difference between pairs of voxels: (A5, A6), (D6, E7) as Figure 9 shows.

### Boosting analysis on the restricted subset

Figure 7 shows that there is still noise weakening the relationship between functional similarities and correlation coefficients within the MFEP specific subset. Therefore, we considered a restricted subset of the MFEP set (consisting of 612 samples) in which the samples have correlation coefficients bigger than 0.7 (illustrated by the square in Figure 7).

Similarly, we applied the boosting analysis on the restricted subset of 612 samples using our proposed weak classifiers and AdaBoost. The dataset was randomly split into two disjoint dataset: a training set (441 samples) and a test set (171 samples). By setting a threshold of 0.67 on the similarity values, there were 35.2% training samples that were assigned label 1, and 30.3% control (test) samples that were assigned label 1.

**Figure 10:**
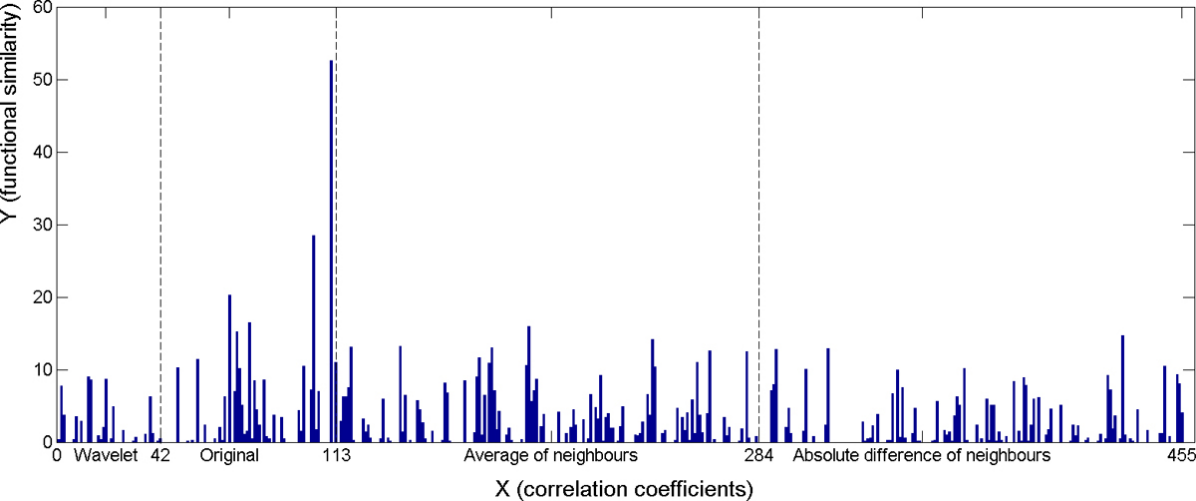
**Cumulated weight of selected features on the restricted subset - Cellular Component.** 1 - 42: wavelet features; 43 - 110: original voxels; 111: the correlation coefficient; 112: the p-value of the correlation coefficients; 113: the Euclidean distance between pair-wise gene maps; 114 - 284: average of neighbouring voxels; 285 - 455: absolute value of difference between neighbouring voxels.

**Figure 11:**
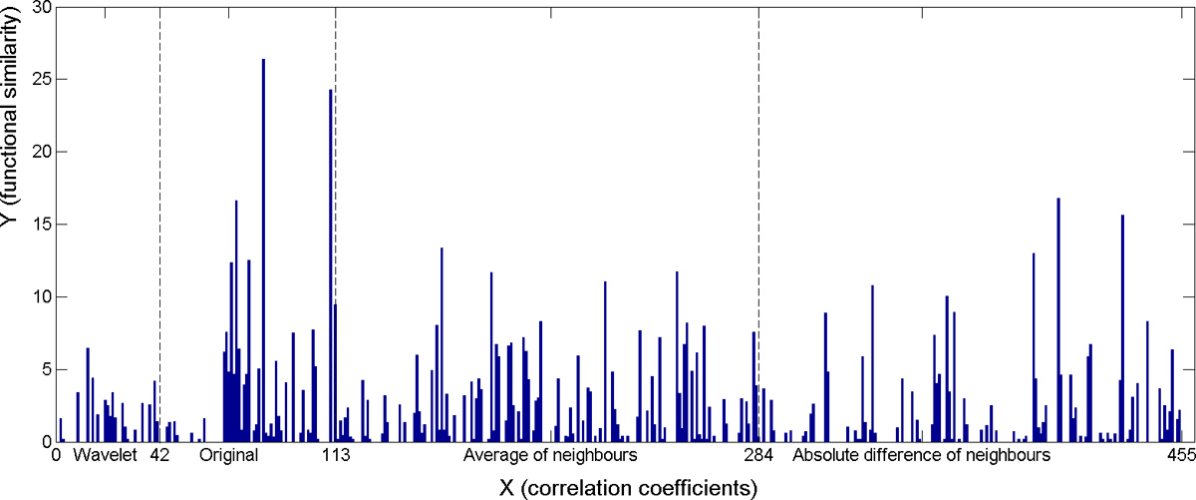
**Cumulated weight of selected features on the restricted subset - Molecular Function.** 1 - 42: wavelet features; 43 - 110: original voxels; 111: the correlation coefficient; 112: the p-value of the correlation coefficients; 113: the Euclidean distance between pair-wise gene maps; 114 - 284: average of neighbouring voxels; 285 - 455: absolute value of difference between neighbouring voxels.

**Figure 12:**
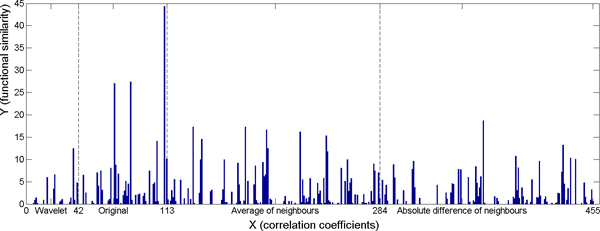
**Cumulated weight of selected features on the restricted subset - Biological Process.** 1 - 42: wavelet features; 43 - 110: original voxels; 111: the correlation coefficient; 112: the p-value of the correlation coefficients; 113: the Euclidean distance between pair-wise gene maps; 114 - 284: average of neighbouring voxels; 285 - 455: absolute value of difference between neighbouring voxels.

There were a total of 455 features for each sample in the experiment. For the weak classifier, we chose the value 20 for the number of bins. For the AdaBoost algorithm, we performed 5000 iterations to reach the best performance of the prediction. Using these settings, we achieved the minimum error on training and control data with respect to all three different gene ontologies (Table 1). The results show that the accuracy of predicting gene functional similarities is better on the restricted subset. Figures 10, 11, 12 show the cumulated weight of selected features of Cellular Component, Molecular Function, and Biological Process respectively. The histogram bar of a certain feature is the sum of the weights of the feature which are selected during the 5000 iterations. Additional file 1 shows the top selected original voxels and the features extracted from neighbouring voxels. Wavelet features were not among the top 10 selected significant features.

### Performing regression by boosting

We applied the proposed Adaboost method to perform regression on the MFEP specific subset of the 59,340 samples. The dataset was randomly split into two disjoint sets: a training set (29,670 samples) and a test set (29,670 samples). The labels of each sample were continuous values in the range 0[1]. The prediction error was calculated using the Root Mean Squared Error (RMSE) of the difference vector between the actual labels and predicted labels. The RMSE was also used in the algorithm to get the error for the strong classifier. There were totally 113 features for each sample in the experiment. For the weak classifier, we chose 100 as the number of bins. For the AdaBoost algorithm, the boosting was repeated for 10,000 iterations to reach a stable performance on the prediction. Using these settings, we calculated the prediction error on the training and control samples. The minimum RMSE on training data and control data with respect to three different gene ontologies are shown in Table 2. By changing the number of regions and number of iterations, the error rate varied. The results indicate that the prediction performance is better on the restricted subset.

**Table 2:** Minimum Root Mean Squared Error (RMSE) on the MFEP specific and restricted subset with respect to gene ontologies

Ontology	RMSE on specific subset		RMSE on restricted subset	
	
	Training data	Control data	Training data	Control data
Cellular Component	0.3287	0.3475	0.2207	0.2829
Molecular Function	0.3928	0.4075	0.2030	0.3582
Biological Process	0.3699	0.3808	0.2371	0.3539

## Discussion

In this study, we identify the pair-wise gene functional similarities by multiplex gene expression maps. This is based on the hypothesis that genes with similar gene expression maps share similar gene functions which was confirmed for a number of genes in previous analysis [14]. Since the dataset we had available only contained gene expression maps, it was hard to use supervised learning to analyze it, so, instead, we built a new dataset in which each sample represented a pair of genes. The features for these samples were the similarity or distance values between two gene expression maps, and the labels were the functional similarities between genes. We used the wavelet transform to extract features from the averaged hemispheres of the mouse brain, and also extracted features from all the pairs of neighbouring voxels. In addition, the correlation coefficients, the p-value of the correlation coefficients and the Euclidean distance were included in the calculation of the difference between gene expression maps. We used the absolute difference between each pair of features of the two genes as the features of samples, and the functional similarities of two genes as the labels. The functional similarities were the averaged function distances for each pair of functions included in the two genes. The similarity (distance) between any two gene functions was obtained by Lin's method based on GO structures. We also built the MFEP specific subset using multiple functional profiles [24] so that the genes in the subset had strong relationship between gene functions and gene expression maps. Based on the MFEP specific subsets, we applied AdaBoost and proposed a weak classifier to fit the characteristics of the dataset. We further restricted the dataset to a more specific one and tested our proposed method on this subset. This methodology can be applied to analyze any large-scale dataset without a target attribute, and is not restricted to gene expressions.

From the experiment on identifying the relationship between similarity of gene expression maps and functional similarity, we observed that with increasing similarities of gene expression maps, the pair-wise genes' functional similarities were also increased, especially for samples with correlation coefficients between pairs of gene maps larger than 0.8. From the boosting analysis, we were able to predict functional similarities of pairs of genes with about 84% accuracy (16% error rate) on the restricted MFEP specific subset. Using the proposed methods, the similarity of pairs MFEP gene expression maps can drive the assignment of new GO terms having similar functions to a new gene.

The selected weak classifiers were able to identify the features that are more important for the prediction. By checking the most selected original features and wavelet features we were able to locate the significant single voxels and the neighbouring voxels in the mouse brain. The most highly selected voxels generally corresponded to the salient neuroanatomical features of the analyzed brain slice. For example, in Figures 8, the most selected voxels corresponded to cortex and striatum. The top selected wavelet features in Additional file 1 also featured cortex and striatum. These observations are consistent with the major molecular and anatomical features of the brain slice.

In the current study, the samples were divided into two classes in accordance to our binary classification formulation. In the future, we plan to use a finer split of the samples (e.g., four or more classes) to improve the precision and model the problem as a regression problem. There are many linear regression algorithms that can handle large amounts of training data. In the future, we will try different regularizers besides boosting, such that there will be no need to make arbitrary thresholds of labels. Furthermore, since the Euclidean distance between wavelet representations may be insufficient to capture non-linearity in the complicated gene map-to-gene function relationship, we plan to investigate other information that is not captured by the wavelet representation. We also plan to incorporate other features besides the current ones into the analysis to further improve the model.

## Competing interests

The authors declare that they have no significant competing financial, professional or personal interests that might have influenced the performance or presentation of the work described in this manuscript.

## Authors' contributions

LA, DJS and VM guarantee the integrity of the entire study. HL and VM conceived the study and contributed in its design and testing. LA designed and implemented the programs. ZO and VM contributed in the design of the experiments. LA, and HL performed the literature research. LA, HL, ZO and VM participated in manuscript preparation, editing and review. DJS provided the biological data and the interpretation of experimental results. All authors read and approved the final manuscript.

## Supplementary Material

Additional file 1 The most selected original voxels on the restricted subset (best viewed in color). Click here for file
