# Supplementary material for: Learning pair-wise gene functional similarity by multiplex gene expression maps
Source: BMC Bioinformatics. 2012 Mar 21;13(Suppl 3):S1. doi: 10.1186/1471-2105-13-S3-S1 (PMC3375633; doi:10.1186/1471-2105-13-S3-S1)
Supplement: Additional file 1 — The most selected original voxels on the restricted subset (best viewed in color). [file 1471-2105-13-S3-S1-S1.pdf]

Supplementary Table 1 - The most selected original voxels on the restricted subset (best viewed in color)

For the features of neighbouring voxels, blue indicates the average expression values of neighbouring voxels; green indicates the absolute value of difference between neighbouring voxels. The darker the mark the more significant the voxel or the pair of voxels is.

<table>
<tr><th>Ontology</th><th>Original voxels</th><th>Features of neighbouring voxels</th></tr>
<tr><td>Cellular
Component</td><td>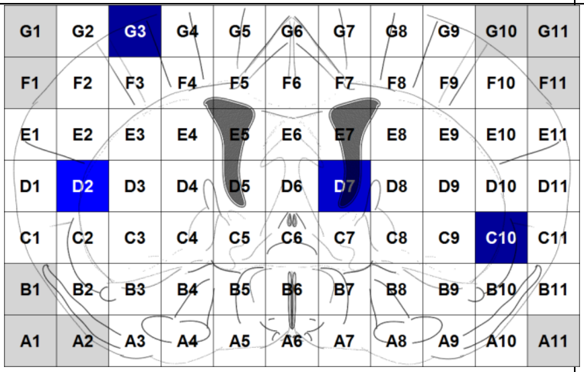
</td><td>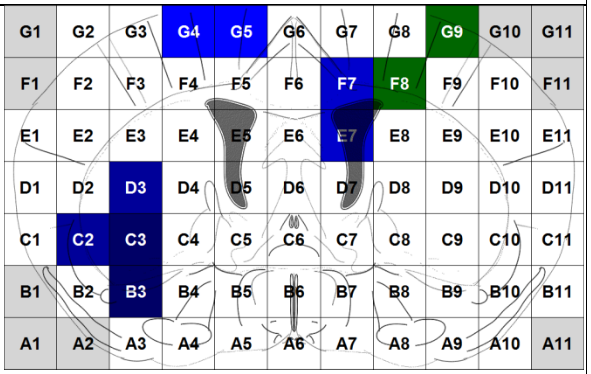
</td></tr>
<tr><td>Molecular
Function</td><td>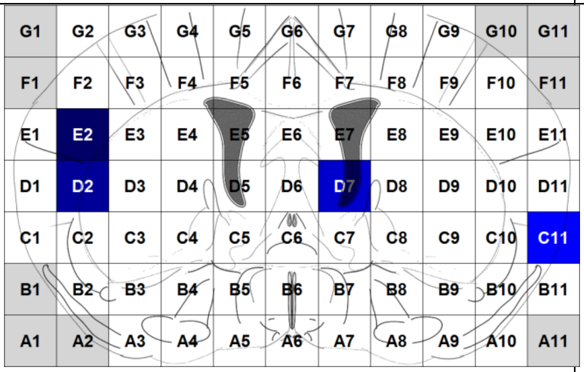
</td><td>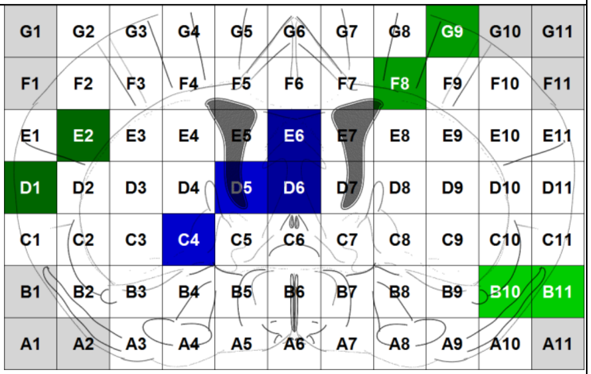
</td></tr>
<tr><td>Biological
Process</td><td>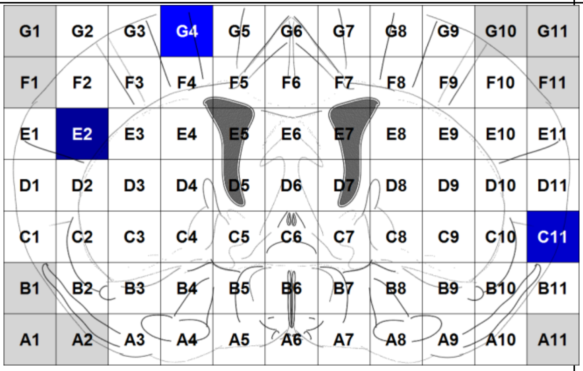
</td><td>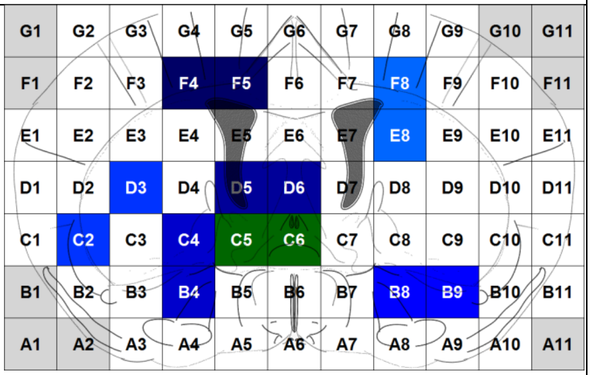
</td></tr>
</table>
